# A City Hospital

**Published:** 1881-04

**Authors:** 


A CITY HOSPITAL.—A building deserving the name, and conducted in a manner commensurate with the interests and reputation of this city, and for the benefit, not only of the poor, but of our whole people, is an urgent necessity at the present time. Baltimore is no longer a village, nor a one horse town, and must own her city hospital, as she cannot afford to lag behind in the march of improvement that is going on in her sister communities in this and in other respects even within her own corporate limits. She cannot, or at least, ought not to be compelled to "farm out" her sick and helpless poor to the lowest bidder, for the want of a hospital for their proper accommodation! Her position in this respect, is an anomalous one, and probably without a precedent in a city of her population and wealth any where! When these facts are considered in connection with her manifestation of public spirit and enterprise in many other directions, the absence of a hospital building, owned and conducted by the city authorities for the benefit of her indigent population, and the treatment of accidents occurring to any of her citizens, is a matter of astonishment; and that she should allow several contemporary municipalities, of even smaller population, to continue so far in advance of her, in the ownership of city hospitals for the objects already named, without putting forth some exertion towards the institution of such a building, is beyond our comprehension! Work will be commenced very soon upon our new Post Office building, and a new Court House is urged, and not without reason, by some of our city papers; but not one word is uttered in behalf of an institution which is loudly called for by our common humanity. The poor are with us, and we cannot escape, if we would, the responsibility of caring for them; and if so, then, let such provision be made for them here as other cities have done for their poor and destitute inhabitants, in the erection and maintenance of properly organized hospitals; and thus, at least, keep abreast of the age and times in which we live? The city should not depend upon the Johns Hopkins' Hospital; even if it were completed, and in active operation; as there would still be an ample field for the exercise of her philanthropy and benevolent care of her indigent population; which should, in no event, be permitted to be provided for alone by the munificence of an individual, however bountiful it might be.

